# Access to emergency hospital care provided by the public sector in sub-Saharan Africa in 2015: a geocoded inventory and spatial analysis

**DOI:** 10.1016/S2214-109X(17)30488-6

**Published:** 2018-01-26

**Authors:** Paul O Ouma, Joseph Maina, Pamela N Thuranira, Peter M Macharia, Victor A Alegana, Mike English, Emelda A Okiro, Robert W Snow

**Affiliations:** a Population Health Unit, Kenya Medical Research Institute (KEMRI)-Wellcome Trust Research Programme, Nairobi, Kenya; b Health Services Unit, Kenya Medical Research Institute (KEMRI)-Wellcome Trust Research Programme, Nairobi, Kenya; c Department of Geography and Environment, University of Southampton, Southampton, UK; d Centre for Tropical Medicine and Global Health, Nuffield Department of Clinical Medicine, University of Oxford, Oxford, UK

## Abstract

**Background:**

Timely access to emergency care can substantially reduce mortality. International benchmarks for access to emergency hospital care have been established to guide ambitions for universal health care by 2030. However, no Pan-African database of where hospitals are located exists; therefore, we aimed to complete a geocoded inventory of hospital services in Africa in relation to how populations might access these services in 2015, with focus on women of child bearing age.

**Methods:**

We assembled a geocoded inventory of public hospitals across 48 countries and islands of sub-Saharan Africa, including Zanzibar, using data from various sources. We only included public hospitals with emergency services that were managed by governments at national or local levels and faith-based or non-governmental organisations. For hospital listings without geographical coordinates, we geocoded each facility using Microsoft Encarta (version 2009), Google Earth (version 7.3), Geonames, Fallingrain, OpenStreetMap, and other national digital gazetteers. We obtained estimates for total population and women of child bearing age (15–49 years) at a 1 km^2^ spatial resolution from the WorldPop database for 2015. Additionally, we assembled road network data from Google Map Maker Project and OpenStreetMap using ArcMap (version 10.5). We then combined the road network and the population locations to form a travel impedance surface. Subsequently, we formulated a cost distance algorithm based on the location of public hospitals and the travel impedance surface in AccessMod (version 5) to compute the proportion of populations living within a combined walking and motorised travel time of 2 h to emergency hospital services.

**Findings:**

We consulted 100 databases from 48 sub-Saharan countries and islands, including Zanzibar, and identified 4908 public hospitals. 2701 hospitals had either full or partial information about their geographical coordinates. We estimated that 287 282 013 (29·0%) people and 64 495 526 (28·2%) women of child bearing age are located more than 2-h travel time from the nearest hospital. Marked differences were observed within and between countries, ranging from less than 25% of the population within 2-h travel time of a public hospital in South Sudan to more than 90% in Nigeria, Kenya, Cape Verde, Swaziland, South Africa, Burundi, Comoros, São Tomé and Príncipe, and Zanzibar. Only 16 countries reached the international benchmark of more than 80% of their populations living within a 2-h travel time of the nearest hospital.

**Interpretation:**

Physical access to emergency hospital care provided by the public sector in Africa remains poor and varies substantially within and between countries. Innovative targeting of emergency care services is necessary to reduce these inequities. This study provides the first spatial census of public hospital services in Africa.

**Funding:**

Wellcome Trust and the UK Department for International Development.

## Introduction

In the past 50 years, health system investments in sub-Saharan Africa have focused on primary care and have substantially increased access to basic curative and preventive services for communicable diseases.^1^ Attention is now increasing for emergency care in Africa, which continues to have high mortality rates from acute maternal and non-communicable diseases.^2^ Emergency care refers to the health system capacity required to ensure effective provision of curative services for life-threatening events.^3^ These events include a diverse set of conditions spanning injuries, obstetrics, and surgical interventions, with the needs of patients being disproportionately highest among low-income and middle-income countries.^4^, ^5^ Without emergency care, many conditions would result in high mortality rates—notably, haemorrhage and hypertensive disorders associated with maternal death.^6^, ^7^ Improvement of quality, access, efficiency, and administration of timely emergency services has been suggested to lead to a 45% reduction in mortality and a 36% reduction in disability in low-income and middle-income countries.^8^

The African Federation for Emergency Medicine, through a series of consensus conferences, highlighted the need to increase access to emergency care via identification of gaps in service provision for targeted interventions.^9^, ^10^ A maximum of 2-h travel times are proposed for emergency hospital care for obstetrics^11^ and emergency surgical interventions.^12^ A 2015 proposal^12^ suggests that at least 80% of any country's population should have access to emergency surgical and anaesthesia services by 2030 to reach international targets of universal health care access to essential medical services.

**Research in context**

**Evidence before this study**

Emergency or acute conditions are becoming major contributors of mortality, contributing to 45% of mortality and 35% of disability in low-income and middle-income countries. They include trauma, surgical, and obstetric conditions, and should normally be handled at higher level facilities called hospitals. Defining access to hospital is therefore crucial for driving the emergency care agenda, particularly with the expected epidemiological shift, where non communicable diseases are expected to be major contributors of mortality in sub-Saharan Africa. However, many countries of sub-Saharan Africa have a paucity of information about the coverage of hospitals, which makes it difficult to measure access gaps. Such a resource can be used in combination with population distribution in a spatial domain to identify inequities in access to care. We searched PubMed and Google Scholar for available literature associated with measuring of geographical access to hospital care in sub-Saharan Africa using the search terms “geographic access”, “access”, “spatial access” and “hospitals”. Our literature searches revealed 30 studies that have defined spatial access to emergency services, such as trauma and surgery in individual countries, but only eight studies in seven countries where analysis was undertaken at national levels were identified, highlighting a paucity of information about geographical access to hospital care for most countries.

**Added value of this study**

We have undertaken, to the best of our knowledge, the first geolocated Pan-African database of public hospitals. We used this list to determine geographical access to hospital care in 48 sub-Saharan African countries and islands, including Zanzibar. Given recommendations for countries to have at least 80% of their population to be living within 2-h travel time of a hospital, this threshold was used to determine populations likely to be geographically marginalised. Our analysis of geographical access revealed substantial gaps in both the total population and women of child bearing age being marginalised from access to public hospital care.

**Implications of all the available evidence**

Physical access to public hospital care is poor in Africa. Sub-Saharan African countries need to develop strategies for bridging these gaps. We show that at least 30 countries need accelerated investments in hospital care to have more than 80% of their respective populations within 2-h travel time of a hospital. Our assembled database provides an important resource that can be easily updated and is made available with this publication. Additionally, the database provides a baseline for future service availability assessments and should be used to undertake more detailed censuses of services provided across hospitals, assess how access might affect trauma, surgical, and obstetric mortality outcomes on the continent, and prioritise investments in emergency care service provision to achieve universal health access by 2030.

To understand within and between country variability in the provision of emergency hospital care, an inventory of hospital service providers and a detailed knowledge of a population's access to these services are required. Hospitals are health facilities expected to provide care for a range of emergency conditions.^13^ Currently, there is no single and spatially defined health facility list for Africa. Although countries have been encouraged by WHO to develop master health facility lists,^14^ most facilities have not been defined with longitudes and latitudes and there is no single Pan-African database of where hospitals are located. Therefore, for the first time in Africa, we aimed to present a composite geocoded assembly of public sector hospital services across the continent south of the Sahara. We use this spatial platform to assess general population access to hospital care metrics in 2015, and specifically for women of child bearing age who would be at the highest risk of maternal mortality if located far from obstetric emergency care.

## Methods

### Assembling of a geocoded inventory

We assembled a geocoded inventory of public hospitals with emergency services in sub-Saharan Africa. In this audit, we focused on the inclusion of public hospitals that are managed by governments at national levels or locally at municipality (eg, in Zimbabwe and South Africa) or local authority (eg, in Kenya and Tanzania), by faith-based organisations, and by non-governmental organisations. In sub-Saharan Africa, these public sector hospitals are often the main service providers of emergency care, especially for rural populations, and are governed by national health policy guidelines and regulations. We excluded private hospitals, which were difficult to audit, although we accept the fact that these hospitals do provide important critical care services for those who can afford them—notably, in urban areas. Additionally, we excluded public hospitals that offer only specialised services (eg, specific psychiatric, leprosy, ophthalmic, spinal, rehabilitative, or tuberculosis facilities). Finally, we excluded services provided to special population groups—notably, military and police service hospitals—and institutional hospitals. Our focus, therefore, is hospital services targeted at a broad range of emergency or referral care to the general population.

We used data from different sources to construct country-specific public hospital lists, including those from ministries of health, health management information systems, and government statistical agencies. We also included data sources from the Humanitarian Data Exchange portal of the UN's Office for the Coordination of Humanitarian Affairs (OCHA) and international organisations such as UNICEF and WHO that assemble facility lists for various purposes. Additionally, we contacted individuals working in various departments of the health ministries for additional sources of master health facility lists that are used for health commodity planning and resource allocation. To ensure there were no duplications of hospitals from the data sources, because countries could list more than one single facility as available on various databases, we cross-checked and reconciled the assembled data (appendix pp 2, 3). We selected hospitals from our data sources on the basis of either the use of the word hospital in the facility name or a level of service provision indicated in the originator lists.

In some databases, the hospital listings were accompanied by longitude and latitude for each facility. However, for those without geographical coordinates, geocoding was required with use of Microsoft Encarta (version 2009), Google Earth (version 7.3), Geonames, Fallingrain, OpenStreetMap, and other national digital gazetteers from national education, census, or statistics departments. We rechecked all coordinates using Google Earth to ensure that the facilities were within the respective country and administrative boundaries of their original lists and were located on major settlements and not on water or offshore.

To cross reference the completeness of our national inventories of public hospitals, we compared our results to the numbers reported in health sector strategic plans (HSSPs), service availability and readiness assessment reports or service provision assessment reports, and the WHO global audit of medical services undertaken in 2014 (appendix pp 2, 3).^15^

### Population data

We obtained estimates of total population and women of child bearing age (15–49 years) at 1 km^2^ spatial resolutions from the WorldPop database for 2015. This population data surface is described elsewhere.^16^ In brief, the most recent census data at the highest administrative unit resolutions used by the national census were disaggregated to land use land cover grids. The different land use land cover classes were assigned weights on the basis of probability of being populated and a random forest technique was used in the disaggregation while adjusting for rural–urban differences and UN urban–rural population growth estimates within each country.^16^ The 1 km^2^ population grids were further disaggregated using additional age and sex sample census and household demographic data to provide estimates of women of child bearing age.^17^

### Road network data

Two sources of road network data for Africa are publicly available: Google Map Maker Project and OpenStreetMap. We combined these data using ArcMap (version 10.5). We included information about major roads that allowed year-round motorised travel, including trunk or highway, primary, secondary, and tertiary or arterial roads. We reclassified these major roads as primary, secondary, and tertiary roads. Those classified as primary were high volume roads that mainly connect international borders. Secondary roads were those that fed into primary roads or connected major towns in different regions of a country. Tertiary roads were those that connected secondary roads while connecting smaller towns or market centres. We corrected the data to connect segments of roads omitted through digitisation and deleted those that extended into water bodies. Each road segment was accompanied by information about average motorised travel speeds and this information was stored as attributes.

### Geographical access

To define geographical access to public hospitals, we used a cost distance algorithm that modelled a composite of walking and motorised travel time to the nearest public hospital. We generated a travel impedance surface by assigning travel speeds of 100 km/h to primary roads, 50 km/h to secondary roads, and 30 km/h to tertiary roads on the basis of previous studies.^18^, ^19^ The model does not give any measures of uncertainty; and for sensitivity analysis, we varied the motorised speeds by more or less than 20% of the original speeds^19^, ^20^ to define an upper and lower bound of uncertainty around travel speeds. We assigned the other non-road raster cells with speeds of 5 km/h, assuming patients could walk, were carried, or were transported with use of other means to the nearest road before obtaining motorised travel.

We used the impedance surface and location of public hospitals to estimate time in hours needed to travel to the nearest public hospital using AccessMod (version 5). The algorithm we used for estimating travel times in AccessMod uses the Manhattan distance, based on travel along road infrastructure, compared with the Euclidean method, which assumes travel in a straight line. Because this algorithm relies on converting roads to a raster surface, the accuracy of the model is in part affected by the spatial resolution. Therefore, we used 100 m spatial grids to capture finer heterogeneity in travel times. Thus, the time needed to get to a hospital is determined by cumulatively adding the time needed to cross the pixels in the so-called least cost path from the location of interest to a hospital. Furthermore, we confined the analysis of travel and accessibility to hospitals to within the national borders, assuming populations do not cross borders to use hospitals in neighbouring countries. We defined national means for island groups (Zanzibar, São Tomé and Príncipe, Cape Verde, and Comoros) by averaging the accessibility quotients from each island's prediction. We computed the proportion of the total population and women of child bearing age within 2 h-travel time to a hospital in AccessMod and mapped results using ArcGIS (version 10.5).

### Data sharing

The full database that supports the findings of this study are available from the Kenya Medical Research Institute Wellcome Trust Research Programme's Population Health page in the Harvard Dataverse, http://DOI:10.7910/DVN/JTL9VY (Ouma P, Okiro EA, Snow RW, 2017), under a CC BY 4.0 license.

### Role of the funding source

The funder of the study had no role in study design, data collection, data analysis, data interpretation, or writing of the report. The corresponding author had full access to all the data in the study and had final responsibility for the decision to submit for publication.

## Results

We consulted 100 databases from 48 sub-Saharan countries and islands (appendix pp 6–22). Zanzibar, which has an independent health system and is governed by a separate ministry of health to mainland Tanzania, was analysed separately. International organisations such as OCHA, WHO, and UNICEF contributed to 33 databases. 31 databases were sourced from ministries of health, nine from the national malaria control programmes, nine from health management information systems, and eight from other ministries and government agencies such as statistical offices. Other sources included journal articles (n=3), web information pages (n=2), unpublished individual documents (n=2), databases from Christian health associations (n=2), and Google Earth (n=1). Of the 100 sources of information, 24 were obtained from personal contacts in ministries of health or non-governmental organisations.

4908 public hospitals were identified across 48 countries and islands of sub-Saharan Africa, including Zanzibar. The numbers of public hospitals likely to be offering emergency or referral care ranged from two in São Tomé and Príncipe to 879 in Nigeria (table). 2701 hospital data sources had either full or partial information about the longitude and latitude, the remaining 2207 hospitals required geocoding. We were unable to geocode 15 public hospitals in Somalia (n=5) and Sudan (n=10). These hospitals were expected to offer services such as surgical care, obstetrics, paediatrics, radiological, and laboratory services (appendix pp 4, 5).

**Table:** Number of public hospitals and access quotients with UI across 48 sub-Saharan African countries and islands, including Zanzibar for 2015

	**Country code (ISO 3)**	**Number of public hospitals**	**Total population in 2015**	**Percentage of population outside 2-h travel time (UI)**	**Total number of women of child bearing age in 2015**	**Percentage of women of child bearing age outside 2-h travel time (UI)**
Angola	AGO	150	21 811 453	36·9% (35·5–39·0)	5 489 490	37·3% (35·8–40·0)
Benin	BEN	48	10 880 616	23·3% (20·9–27·2)	2 136 501	23·7% (21·3–27·6)
Botswana	BWA	29	2 258 625	23·3% (20·9–27·5)	622 953	23·3% (20·9–27·5)
Burkina Faso	BFA	62	18 069 713	46·9% (44·8–50·0)	4 091 918	45·2% (43·1–48·2)
Burundi	BDI	49	11 162 902	4·3% (3·9–5·0)	2 515 577	4·2% (3·8–4·9)
Cameroon	CMR	184	23 342 359	17·4% (16·4–18·8)	5 230 085	17·1% (16·3–18·7)
Cape Verde	CPV	9	488 986	6·6% (6·6–6·6)	132 377	7·0% (6·9–7·0)
Central African Republic	CAF	20	4 898 576	51·5% (47·3–55·6)	1 192 470	51·6% (47·4–55·7)
Chad	TCD	78	14 022 236	53·1% (51·4–55·7)	2 973 342	53·2% (51·4–55·8)
Comoros	COM	3	697 609	3·4% (2·4–5·8)	173 474	3·5% (2·5–6·0)
Congo (Brazzaville)	COG	25	4 584 395	27·7% (26·6–29·1)	1 186 661	27·8% (26·7–29·1)
Côte d'Ivoire	CIV	100	22 699 552	34·4% (32·9–36·8)	5 659 963	34·4% (32·9–36·8)
Democratic Republic of the Congo	COD	435	72 001 218	46·3% (44·0–49·2)	14 330 432	46·2% (43·9–49·2)
Djibouti	DJI	13	870 713	16·7% (16·1–17·6)	211 489	17·0% (16·4–17·9)
Equatorial Guinea	GNQ	18	824 276	24·2% (22·2–27·4)	188 338	24·2% (22·3–27·3)
Eritrea	ERI	22	5 210 651	57·4% (55·5–59·8)	1 345 007	55·7% (53·9–58·0)
Ethiopia	ETH	161	99 337 653	49·3% (45·5–54·3)	22 721 668	49·3% (45·4–54·2)
Gabon	GAB	59	1 628 849	16·4% (15·9–17·0)	397 063	16·4% (16·0–17·1)
The Gambia	GMB	6	1 950 904	28·5% (28·1–29·3)	411 783	29·3% (28·9–30·0)
Ghana	GHA	178	27 098 194	13·8% (12·8–15·5)	7 126 334	13·9% (12·8–15·6)
Guinea	GIN	35	12 546 646	37·3% (35·0–40·3)	2 520 496	37·2% (34·9–40·2)
Guinea-Bissau	GNB	8	1 745 803	38·5% (34·6–44·4)	429 740	38·4% (34·5–44·3)
Kenya	KEN	399	45 737 778	7·1% (6·5–8·1)	11 243 809	7·1% (6·4–8·0)
Lesotho	LSO	20	2 136 640	43·3% (40·8–46·4)	570 583	43·3% (40·9–46·5)
Liberia	LBR	38	4 451 499	38·5% (36·9–40·7)	1 090 502	38·5% (36·9–40·6)
Madagascar	MDG	125	24 120 532	53·4% (52·0–55·8)	5 262 812	53·3% (51·9–55·7)
Malawi	MWI	56	17 207 197	7·2% (5·5–10·2)	3 938 576	7·2% (5·5–10·2)
Mali	MLI	76	17 619 152	36·2% (33·3–40·6)	3 258 813	35·3% (32·4–39·7)
Mauritania	MRT	18	4 026 075	61·4% (59·1–64·1)	880 617	61·4% (59·1–64·2)
Mozambique	MOZ	61	27 673 736	49·9% (44·8–52·7)	6 431 717	49·8% (44·6–52·5)
Namibia	NAM	35	2 461 440	23·2% (20·2–27·4)	644 284	23·2% (20·3–27·5)
Niger	NER	41	19 805 985	57·2% (53·6–61·7)	3 376 284	57·1% (53·5–61·6)
Nigeria	NGA	879	182 178 061	7·7% (6·9–9·0)	43 659 033	8·5% (7·8–10·1)
Rwanda	RWA	47	11 585 862	11·2% (10·6–12·1)	2 793 168	11·3% (10·7–12·2)
São Tomé and Príncipe	STP	2	186 623	2·7% (2·4–3·1)	43 894	2·7% (2·5–3·2)
Senegal	SEN	29	14 967 332	39·7% (36·2–41·2)	3 583 623	39·6% (36·1–41·1)
Sierra Leone	SLE	32	6 418 015	39·6% (36·9–39·1)	1 537 021	39·6% (37·0–39·2)
Somalia	SOM	79	10 688 048	44·0% (42·5–47·4)	2 179 717	42·3% (40·9–45·6)
South Africa	ZAF	327	54 345 833	5·2% (4·4–6·3)	15 021 723	5·2% (4·5–6·4)
South Sudan	SDS	40	12 347 507	77·2% (76·5–79·0)	2 786 192	77·3% (76·6–79·0)
Sudan	SDN	272	40 249 394	53·8% (53·2–56·5)	9 346 646	53·7% (53·1–56·3)
Swaziland	SWZ	7	1 285 392	6·1% (3·7–8·9)	289 274	6·1% (3·8–9·0)
Tanzania (mainland)	TZA	210	53 265 074	24·9% (22·1–29·0)	12 585 409	24·8% (22·1–29·0)
Togo	TGO	38	7 304 010	14·7% (12·9–17·4)	1 588 063	14·8% (13·3–17·7)
Uganda	UGA	121	39 032 494	17·5% (15·5–20·7)	7 979 425	17·5% (15·5–20·8)
Zambia	ZMB	91	16 218 094	40·1% (37·1–43·7)	3 699 046	40·0% (37·0–43·7)
Zanzibar	··	4	1 579 927	2·7% (2·0–5·6)	376 652	2·6% (1·9–5·5)
Zimbabwe	ZWE	169	15 604 001	20·7% (18·6–23·9)	3 453 496	21·5% (19·4–24·6)
Total	··	4908	990 627 630	29·0% (27·1–31·5)	228 707 540	28·2% (26·4–30·8)

UI=uncertainty intervals.

It was possible to compare the summaries of hospital services in 30 countries, where these were specified in both the HSSPs and the 2014 WHO audit.^15^ Overall, the number of hospitals audited during the present exercise (n=3203) and HSSPs (n=3469) were broadly comparable, but greater than those specified in the 2014 WHO audit (n=2518). There were substantial differences in the numbers of hospitals reportedly available in Ethiopia (212 hospitals audited from HSSPs, 187 from WHO, and 161 from present audit), Sudan (428 from HSSP, 255 from WHO, and 262 from present audit), and Uganda (160 from HSSP, 64 from WHO, and 121 from present audit).

**Figure 1:**
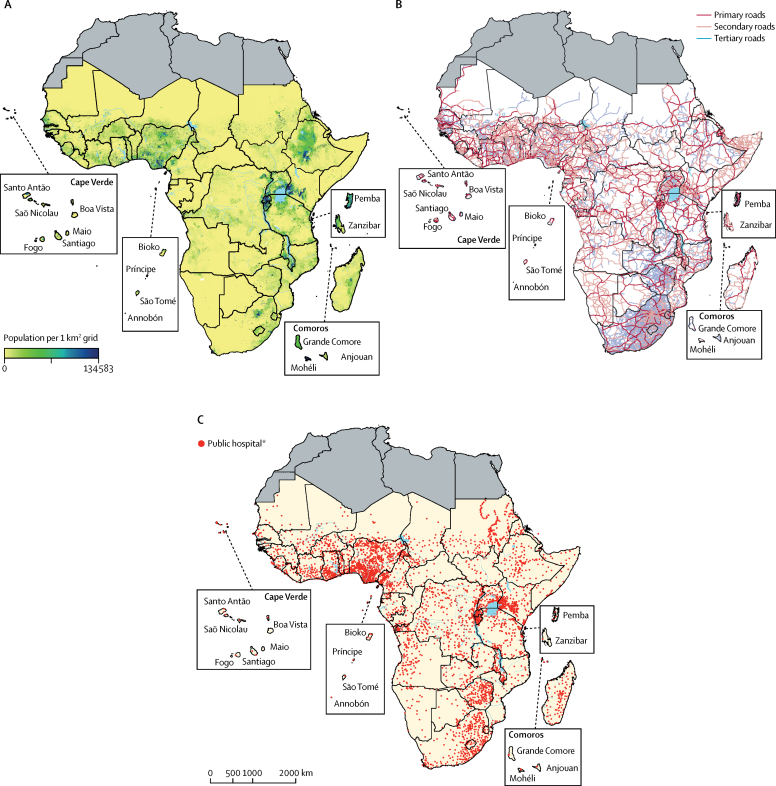
Population density, road network coverage, and locations of public hospitals in sub-Saharan Africa in 2015 Regions shaded in grey were not included, as they are not part of sub-Saharan Africa. (A) Population density per 1 km^2^. (B) Coverage of road network where motorised travel to hospitals is possible. (C) Distribution of 4893 public hospitals. *15 hospitals could not be geocoded.

**Figure 2:**
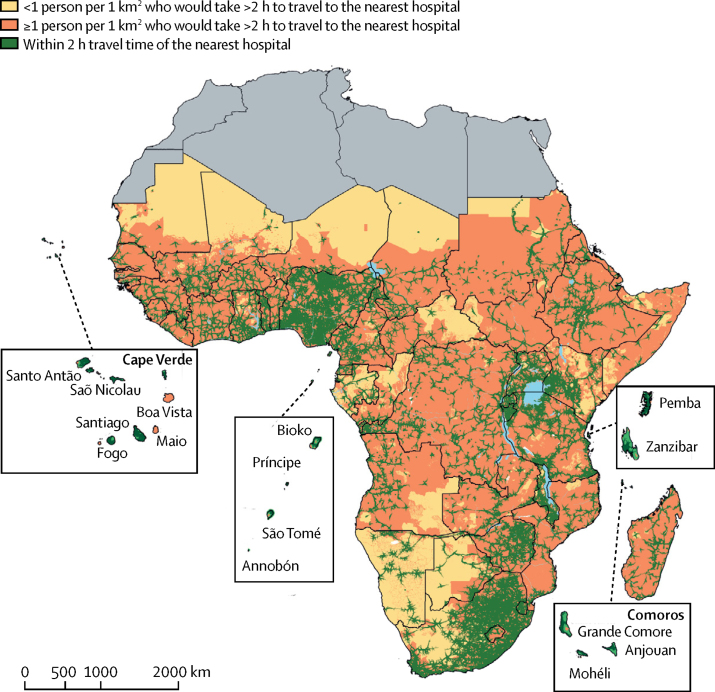
Geographical access of the general population to public hospitals Regions shaded in grey were not included.

As expected, where population density was high, hospital density was also high (figures 1A, 1C). 703 345 617 (71%) people and 164 212 014 (71·8%) women of child bearing age were living within 2-h travel time of the nearest public hospital across the 48 sub-Saharan African countries or offshore islands (table). Conversely, the most geographically marginalised in 2015 comprised 287 282 013 (29%) people and 64 495 526 (28·2%) women of child bearing age who had travel times more than 2 h from emergency public hospital care. Populated areas with more than one person per km^2^ but outside the 2-h motorised travel time to public hospital care are shown in figure 2 to highlight areas in need of greatest service accessibility.

**Figure 3:**
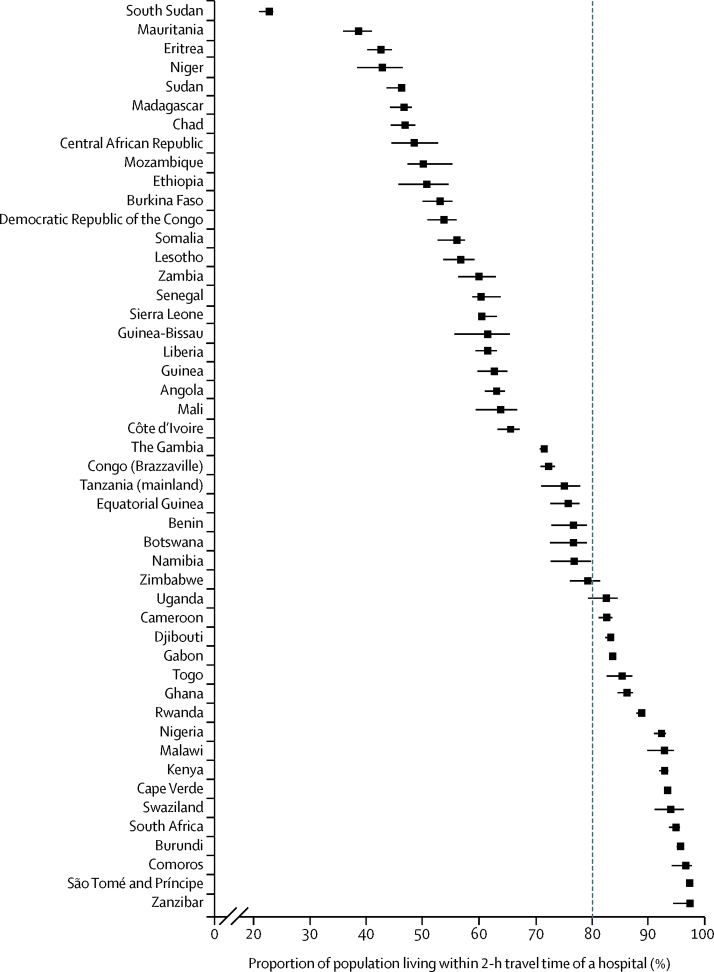
Proportion of population living within 2-h travel time of a hospital in 2015 in sub-Saharan Africa Error bars are uncertainty intervals. The dotted line distinguishes between countries that have 80% of their populations within 2-h travel time of a hospital and those yet to achieve this proportion.

Geographical accessibility to emergency hospital care varied between countries (table, figure 3), ranging from less than 25% of the population within 2-h travel time of a public hospital in South Sudan to more than 90% in Nigeria, Kenya, Cape Verde, Swaziland, South Africa, Burundi, Comoros, São Tomé and Príncipe, and Zanzibar. Seven countries had less than 50% of the population within 2-h travel time of a public emergency care hospital: South Sudan, Mauritania, Eritrea, Niger, Sudan, Madagascar, and Chad. Several large countries such as South Sudan, Mauritania, Democratic Republic of the Congo, Mozambique, and Zambia have poor access to hospital care compared with smaller countries or islands such as Cape Verde, Zanzibar, and São Tomé and Príncipe. However, notable exceptions were observed with large countries such as Nigeria, Kenya, and South Africa having more than 90% of their respective population living within 2-h travel time of a hospital, whereas smaller countries such as Eritrea and Lesotho have poor access. Only 16 countries met the international recommendation^12^ of more than 80% of the population within 2-h travel time of a hospital (figure 3).

## Discussion

We have assembled the first Pan-African, geocoded database of public hospitals in 48 countries and islands. We estimated that in Africa, about 29% of the population and about 28% of women of child bearing age are geographically marginalised from emergency medical, obstetric, and surgical care, and live more than 2-h travel time to the nearest public hospital; and only 16 of 48 countries have more than 80% of their population living within 2-h travel time of emergency hospital care. We note that smaller countries and islands have proportionately better access to hospital services than in larger countries, where more than 40% of the population live more than 2-h travel time to a public hospital. However, we noted exceptions where large countries have better access quotients than in smaller countries. One important function of routine hospital services is the ability to provide emergency obstetric care, and we have defined access to hospital care for both women of child bearing age and total populations. To reach geographically marginalised populations with hospital care, innovative targeting of emergency care is required, including improvement of transportation modes, ambulatory services, or the numbers of hospitals in specific geographical locations.

Health facility lists in Africa are fragmented, only 31 (31%) of 100 original sources used in this study were from ministries of health, and a diverse list of sources, especially from other governmental and international agencies, were required to provide a more comprehensive understanding of hospital care. The accuracy and completeness of this resource now requires further country and regional level efforts, although our initial database serves as a useful entry point to future hospital censuses in Africa. There was no universal definition of hospital or emergency care, and definitions provided in national health policies varied between countries (appendix pp 4, 5). This absence of a definition has been noted previously and demands a more standard classification of emergency hospital care provision in Africa.^21^ We were not able to ascertain what services are provided at each of the mapped public hospitals. Additionally, the number of hospitals audited during the present exercise differed to those specified in the HSSPs and 2014 WHO audit of specific countries, possibly as a result of differences in years for which an HSSP was published or inclusion or exclusion of private sector hospitals where this number was not made clear in the HSSP. A review of 48 HSSPs, the cornerstone policy documents that describe the national health system, highlights the inadequacy of national definitions of hospital services, minimum clinical and laboratory equipment, or staffing needs that should constitute this higher level of the health sector (appendix pp 4, 5). The actual level of service provision should in theory be available from national surveys of service availability and readiness assessment reports and service provision assessment reports;^22^ however, these surveys are often only sampled facilities and have only been undertaken in 18 sub-Saharan countries.^23^

The role of the private sector in achieving universal health coverage is essential,^24^ but it remains poorly enumerated in sub-Saharan Africa, including our audit, despite extensive searches from multiple sources. Future hospital service censuses and audits must include the private sector because these services compete with public sector services notably in urban areas.

Our accessibility analysis had a number of limitations. We were unable to evaluate the efficiency, timeliness, and abilities of referral transport systems in each country with adequate precision at a continental scale. We have not been able to account for frequency of transport services on secondary to main roads connected to hospital locations, the precise transport speeds, how prostrated emergency care patients are transported from households to arterial road networks, or the multitude of other physical and financial barriers to referral from home to hospital in each country or subregions. Additionally, we were unable to account for dynamic population changes at very fine spatial resolutions because these data are unavailable at continental scales. These additional analyses are beyond the scope of the present paper, but should be highlighted to build knowledge across the extensive domains of hospital service access through increased spatial resolution studies^25^, ^26^ and understanding of referral care for hospital services in Africa.

In summary, consensus on the need to integrate emergency care into health systems is increasing.^27^, ^28^ Key towards addressing challenges in emergency care is defining access to hospitals and highlighting populations most distal from these services. We have assembled the first geocoded database of public hospitals in sub-Saharan Africa, and have used this audit or inventory to provide a ranking of the worst and best hospital-served countries that in theory should be able to provide vital emergency services for trauma, surgical, and obstetric patients. Most countries were well below the benchmark set for 2030, where less than 80% of the population lived within 2-h travel time of emergency hospital care. The importance of hospital services goes beyond emergency care, hospitals additionally provide the core backbone to surveillance of emerging or escalating antimicrobial resistance, the detection of new pathogen epidemics, and provide the means to define the operational effectiveness of the introduction of new vaccines or other community-based interventions. Hospitals provide an essential part of pathogen and intervention effect across Africa, and knowing where they are located is essential. Definition of the scope, service provision capacities, laboratory capacities, and optimal catchment populations for emergency hospital care should be a priority. Ultimately, we provide a resource to begin these urgent censuses of hospital services in Africa and where applications extend beyond just defining physical access.
