# Supplementary material for: Access to emergency hospital care provided by the public sector in sub-Saharan Africa in 2015: a geocoded inventory and spatial analysis
Source: Lancet Glob Health. 2018 Jan 26;6(3):e342–50. doi: 10.1016/S2214-109X(17)30488-6 (PMC5809715; doi:10.1016/S2214-109X(17)30488-6)
Supplement: Supplementary appendix [file mmc1.pdf]

THE LANCET
Global Health

Supplementary appendix

This appendix formed part of the original submission and has been peer reviewed. We post it as supplied by the authors.

Supplement to: Ouma PO, Maina J, Thuranira PN, et al. Access to emergency hospital care provided by the public sector in sub-Saharan Africa in 2015: a geocoded inventory and spatial analysis. *Lancet Glob Health* 2018; published online Jan 24. http://dx.doi.org/10.1016/S2214-109X(17)30488-6.

Supplementary Appendix

Table 1 is a comparison of our assembled hospital numbers with those reported by health sector strategic plans and the WHO per country. The hospital numbers are shown in Table 1, which also captures the year each source represents. We mapped hospitals in 48 countries, while from HSSPs and WHO, we could identify number of hospitals in 41 and 33 countries respectively. In 30 countries, we had hospital numbers from all the three sources.

Table 2 shows the specific minimum essential services that should be available at first level referral hospitals for each country. The catchment population expected to be served by these hospitals are also shown. The description of essential services was available for 32 countries while catchment populations could only be defined for 21 countries.

Table 1: Description and comparison of hospital numbers from health sector strategic plans, WHO and our assembled databases, including the estimated year each source represents.

Country	Number of public hospitals (HSSP) [Ref]	WHO Number of public hospitals [1]	No. Mapped/Tot public hospitals & Est. Dates	Source of mapped hospitals
Angola	209 (2012-2025) [2]	NR	150/150 (2013)	[3,4]
Benin	36 (2009-2018) [5]	42	48/48 (2014)	[6,7]
Botswana	26 (2011) [8]	26	29/29 (2014)	[9]
Burkina Faso	52 (2011-2020) [10]	50	62/62 (2007)	[11,12]
Burundi	69 (2016-2025) [13]	51	49/49 (2014)	[14,15]
Cameroon	176 (2016-2025) [16]	170	184/184 (2015)	[17,18]
Cape Verde	NR (2012-2016) [19]	5	9/9 (2012)	[20,21]
Central African Republic	NR (2015-2016) [22]	22	20/20 (2013)	[23]
Chad	NR (2013-2015) [24]	77	78/78 (2013)	[25,26,27]
Comoros	3 (2010-2014) [28]	5	3/3 (2004)	[29]
Congo	31 (2007-2011) [30]	NR	25/25 (2013)	[31]
Côte d'Ivoire	85 (2013-2015) [32]	86	100/100 (2013)	[33,34]
Democratic Rep of Congo	497 (2011-2015) [35]	301	435/435 (2014)	[36,37,38]
Djibouti	NR (2013-2017) [39]	NR	13/13 (2016)	[40]
Equatorial Guinea	NA	NR	18/18 (NA)	[41]
Eritrea	25 (2012-2016) [42]	22	22/22 (2013)	[43]
Ethiopia	212 (2016-2020) [44]	187	161/161 (2011)	[45,46,47,48]
Gabon	58 (2011-2015) [49]	55	59/59 (2012) [a]	[50,51]
Gambia	5 (2012-2020) [52]	13	6/6 (2015)	[53,54]
Ghana	199 (2014-2017) [55]	32	178/178 (2016)	[56]
Guinea	41 (2015-2024) [57]	41	35/35 (2015)	[58,59]
Guinea-Bissau	NR (2008-2017) [60]	NR [b]	8/8 (2017) [c]	[61]
Kenya	359 (2013-2017) [62]	260	399/399 (2016)	[63,64,65,66]
Lesotho	19 (2012-2017) [67]	NR	20/20 (2012)	[68,69]
Liberia	26 (2011-2021) [70]	16	38/38 (2014)	[71,72]
Madagascar	125 (2015-2019) [73]	54	125/125 (2012)	[74,75]
Malawi [d]	48 (2011-2016) [76]	65	56/56 (2016)	[77,78,79,80]
Mali [e]	70 (2014-2023) [81]	70	76/76 (2015)	[82,83]
Mauritania	13 (2012-2020) [84]	20	18/18 (2014)	[85]
Mozambique	51 (2001-2010) [86]	NR	61/61 (2013)	[87,88,89]
Namibia	33 (2010-2020) [90]	34	35/35 (2009)	[91,92,93]
Niger	45 (2013-2020) [94]	54	41/41 (2014)	[95,96]
Nigeria	950 (2010-2015) [97]	NR	879/879 (2012)	[98]
Rwanda	45 (2012-2018) [99]	NR	47/47 (2016)	[100,101]
São Tomé and Príncipe	NR (2012-2015) [102]	NR	2/2 (2011)	[103]
Senegal	20 (2009-2018) [104]	22	29/29 (2012)	[105]
Sierra Leone	41 (2010-2015) [106]	NR	32/32 (2015)	[107,108]

Country	Number of public hospitals (HSSP) [Ref]	WHO Number of public hospitals [1]	No. Mapped/Tot public hospitals & Est. Dates	Source of mapped hospitals
Somalia	55 (2013-2016) [109,110,111]	NR	74/79 (2015)	[112,113,114,115]
South Africa	315 (2015 - 2020) [116]	356	327/327 (2014)	[117]
South Sudan	37 (2012-2016) [118]	NR	40/40 (2015)	[119]
Sudan	428 (2003-2027) [f] [120]	255	262/272 (NA)	[121]
Swaziland	8 (2014-2018) [122]	4	7/7 (2010)	[123]
Tanzania (mainland)	208 (2015-2020) [124]	NR	210/210 (2015)	[125]
Togo	44 (2012-2015) [126]	41	38/38 (2013)	[127,128,129,130,131]
Uganda	160 (2015-2020) [132]	64	121/121 (2017)	[133,134,135]
Zambia	109 (2011-2015) [136]	57	91/91 (2012)	[137]
Zanzibar	6 (2007-2011) [138]	NR	4/4 (2013)	[139,140]
Zimbabwe	182 (2016-2020) [141]	65	169/169 (2009)	[142,143,144,145]

a. In Gabon, Centres médicaux are included as first reference hospitals
b. WHO number of hospitals in Guinea Bissau were excluded because it reports an unrealistic number of 962.
c. The number of hospitals in Guinea Bissau were only checked with a published report [146] that is not directly from the MoH
d. In Malawi, rural/community hospitals were excluded as they only offer primary level services
e. In Mali, referral health centres are included as district hospitals
f. Number of hospitals reported in Sudan HSSP could be inclusive of South Sudan because HSSP was formulated in 2003, long before South Sudan became a republic.

Abbreviations for data sources

HSSP: Health Sector Strategic Plan; **NR:** No Record; **WHO:** World Health Organization

Table 2: Sources of data on catchment populations served by primary hospitals and the minimum essential services the first level referral hospitals provide.

Country	Source	Catchment population for first level referral hospitals	Specific minimum essential services
Angola	[2,147]	150,000 - 500,000	Laboratory, in-patient care, radiology
Benin	[5]	NR	NR
Botswana	[8,148]	NR	Obstetrics, gynecology, psychiatric care, oncology
Burkina Faso	[10,149]	NR	Surgery, appendicitis
Burundi	[13]	NR	NR
Cameroon	[16]	NR	NR
Cape Verde	[19]	NR	NR
Central African Republic	[22]	NR	NR
Chad	[24]	NR	Surgery, obstetrics, medicine
Comoros	[28]	NR	NR
Congo	[30]	NR	Surgery, obstetrics, pediatrics, medicine
Côte d'Ivoire	[32]	NR	NR
Democratic Rep of Congo	[35]	100,000-200,000	Surgery, obstetrics, pediatrics, medicine, gynecology
Djibouti	[39]	NR	NR
Equatorial Guinea	NR	NR	NR
Eritrea	[42]	50,000-100,000	Surgery, obstetrics
Ethiopia	[44,151]	60,000-100,000	Surgery, blood transfusion
Gabon	[49]	NR	NR
Gambia	[52]		Surgery, obstetrics, blood transfusion
Ghana	[55,151]	100,000–200,000	Surgery, obstetrics, gynecology, child health, medicine, anesthesia, accident, emergency services
Guinea	[57]	Prefecture population	Surgery, obstetrics, pediatrics, medicine, gynecology
Guinea Bissau	[60]	NR	NR
Kenya	[62,152]	100,000	Surgery, inpatient care, blood transfusion, laboratory, consultative services
Lesotho	[67]	District population	Specialized services, laboratory services
Liberia	[70]	200,000	Surgery, obstetrics, pediatrics, general medicine, gynecology
Madagascar	[73]	District population	Surgery, obstetrics, pediatrics, medicine, neonatal care
Malawi	[76]	District population	Surgery, trauma care
Mali	[81]	250,000	NR
Mauritania	[84]	NR	NR
Mozambique	[86]	50,000-250,000	Surgery, radiology, emergency services
Namibia	[90,153]	NR	Surgery, obstetrics
Niger	[94,154]	District population	Inpatient services, caesarean sections
Nigeria	[97]	NR	Surgery, obstetrics, gynecology, pediatrics
Rwanda	[99,155]	District population	Surgery, obstetrics, gynecology, inpatient/outpatient services, laboratory, radiology
São Tomé and Príncipe	[102]	NR	
Senegal	[104]	NR	Surgery, general medicine
Sierra Leone	[106]	NR	Inpatient and diagnostic services, management of accidents, emergencies

Country	Source	Catchment population for first level referral hospitals	Specific minimum essential services
Somalia	[109,110,111]	NR	NR
South Africa	[116]	NR	Surgery, obstetrics and gynecology, pediatrics, trauma care, family medicine, radiology, anesthetics
South Sudan	[118]	300,000	Surgery, obstetrics, pediatrics, medicine
Sudan	[120]	NR	NR
Swaziland	[122]	NR	Surgery, ear, nose and throat (ENT), ophthalmology, dentistry, intensive care, radiology, pathology
Tanzania (mainland)	[124,156]	250,000	Surgery, Medicine
Togo	[126]	NR	NR
Uganda	[132,157]	100,000-500,000	Surgery, obstetrics and gynecology, medicine, pediatrics, family medicine, X-ray
Zambia	[136]	80,000-200,000	Surgery, obstetrics, medicine and diagnostic services
Zanzibar	[138,158]	District population	Surgery, obstetrics, laboratory testing, radiology
Zimbabwe	[141]	140,000	Comprehensive preventive, curative services

Catchment population and hospital services limited to only the first level referral hospitals
Somalia represents the three regions of Puntland, Somalia and Somaliland

Abbreviations

NR: No record

References

1. WHO (2014). Country data - Global atlas of medical devices. Accessed April 21, 2017 at [http://www.who.int/medical_devices/countries/en/]

2. Ministério da saúde [Angola] (2012). Plano Nacional de Desenvolvimento Sanitario 2012-2025. Luanda, Angola. Accessed April 1, 2017 at [http://www.minsa.gov.ao/VerPublicacao.aspx?id=1266]

3. World Learning (2013). Health facility listing for eight Angolan provinces. Unpublished database provided by Fern Teodoro and Fernando David on December 18, 2013

4. Ministério da Saúde (2014). Compilation of Health Statistics of the National Health System for the year 2014. Unpublished data provided by Ernest Dabire on July 26, 2017 and List of health facilities in the provinces of Huila, Cunene and Namibe. Unpublished excel database provided by John Mendhelsohn on April 21, 2017

5. Ministere de la sante [Benin] (2009). Plan National de Développement Sanitaire 2009-2018. Porto Novo, Benin. Accessed April 1, 2017 at [http://www.sante.gouv.bj/documents/PNDS.pdf]

6. United Nations Population Fund (2014). Facilities for monitoring Emergency Obstetrics Neonatal Care in Benin. Unpublished excel database provided by Ahanhanzo Cesaire Damien on March 1 and 4, 2014

7. Benin Système National d'Information et de Gestion Sanitaires. Accessed at [https://dhis-bj.org/dhis-web commons/security/login.action]

8. Ministry of Health [Botswana] (2011). National Health Policy 2011. Gaborone, Botswana. April 12, 2017 at [http://www.moh.gov.bw/Publications/policies/revised_National_Health_Policy.pdf]

9. Health Information Management System Division, Master Health Facility List, Botswana. Accessed March 5, 2015 at [http://www.moh.gov.bw/Publications/tollfree%20numbers.pdf]

10. Ministere de la sante [Burkina Faso] (2011). Plan National de Développement Sanitaire 2011-2020. Ouagadougou, Burkina Faso. April 12, 2017 at [https://www.internationalhealthpartnership.net/fileadmin/uploads/ihp/Documents/Country_Pages/Burkina_Faso/Burkina_Faso_National_Health_Strategy_2011-2020_French.pdf]

11. Ministere de la Sante [Burkina Faso] (2008). Carte Sanitaire 2007. Unpublished database from provided by Clarisse Bougouma on December 13, 2012

12. Programme National de Lutte contre le Paludisme [Burkina Faso] (2013). Burkina Faso health facilities. Unpublished database provided by Wilfried Koadio Ouattara on May 5, 2012

13. Ministere de la sante (2016). Politique Nationale de Sante 2016-2025. Bujumbura, Burundi. April 12, 2017 at [https://www.minisante.bi/images/Documents/PNS%202016%202025%20VF%2021052016.pdf]

14. Ministere de la Sante Publique et de Lutte contre le Sida, Institut National de Sante Publique INSP, Banque Mondiale (2014). Evaluation de la Qualite des Prestations dans les Formations Sanitaires du Burundi. Rapport Definitif. 2ème Edition. Accessed September 14, 2016 at [http://tinyurl.com/o6yzedz]

15. Ndayizeye F, Niyonkuru D, Ngoga E (2015). Rapport d'enquête sur la disponibilité, l'accessibilité, l'utilisation des intrants de lutte contre le paludisme ainsi que la qualité de prise en charge du paludisme dans des structures sanitaires sélectionnées.

16. Ministère de la Santé Publique [Cameroon] (2011). Plan National de Developpement Sanitaire 2016-2025. Yaoundé, Cameroon. Accessed April 3, 2017 at [https://www.internationalhealthpartnership.net/fileadmin/uploads/ihp/Documents/Country_Pages/Cameroon/Cameroon_National_Health_Plan_2011-2015_French.pdf]

17. Ministere de la Sante Publique [Cameroun] (2011), Cartographie des Formations Sanitaires au Cameroun 2011. Accessed April 22, 2014 at [http://tinyurl.com/luv75jy]

18. Ministere de la Sante Publique [Cameroun]. Cameroun health management information system health facilities. Excel database provided by Kuetche Magloire Takougang and Dorothy Achu on July 11, 2014

19. Ministério da Saúde [Cape Verde] (2012). Plano Nacional de Desenvolvimento Sanitario II 2012-2016. Praia, Cape Verde. Accessed April 3, 2017 [http://www.nationalplanningcycles.org/sites/default/files/planning_cycle_repository/cape_verde/cabo_verde_pnds_vol_i_2012_2016_versao_final_01_100313.pdf]

20. Ministerio da Saude (2012). Plano Nacional de Desenvolvimento Sanitario 2012 – 2016. Ministerio da Saude, Republica de Cabo Verde. Accessed on September 7, 2016 at [http://tinyurl.com/hh7ns2d]

21. Cabo Verde Info (2015). Saude e Bem Estar, Sociedade. Accessed on September 6, 2016 at [http://tinyurl.com/j3pzdwt]

22. Ministere de la sante [Central Africa Republic] (2015). Plan De Transition Du Secteur Santé En République Centrafricaine 2015-2016. Bangui, Central Africa Republic. Accessed April 3, 2017 at [http://www.nationalplanningcycles.org/sites/default/files/planning_cycle_repository/central_african_republic/rca_-ptss_v_definitive_1.pdf]

23. Roll Back Malaria Central African Regional Network (2014). A list of registered health facilities in Central African Republic. Unpublished database provided by José Nkuni on August 3, 2014.

24. Ministère de la Santé Publique [Chad] (2013). Plan National De Developpement Sanitaire 2013-2015. N'Djamena, Chad. Accessed April 4, 2017 at [https://www.google.com/url?sa=t&rct=j&q=&esrc=s&source=web&cd=1&cad=rja&uact=8&ved=0ahUKEwi4nvqd3OTTAhVBKywKHaN3BokQFggmMAA&url=http%3A%2F%2Fwww.sante-tchad.org%2Ffile%2F151097%2F&usg=AFQjCNH9vM7OVg8UFTlqn_IJ-aOA00uf1Q&sig2=XHTuQNmG0SSbg2o3Yye-RA]

25. Ministère de la Santé Publique [Tchad] (2013). Annuaire des Statistiques Sanitaires 2013. Tome A 27ème Edition Annee 2013

26. Programme National de Lutte contre le Paludisme au Tchad (2012). Health Facilities in Chad. Unpublished data provided by Jose Nkuni, Djoumbe Ephraïm and Clément K. Hinzoumbé on December 20, 2013

27. Office for the Coordination of Humanitarian Affairs (2013). Geodatabase with GIS data and statistics data for Chad including boundaries, transportation, hydrology, health facilities, population data. Accessed May 13, 2013 at [https://data.humdata.org/dataset/chad-gis-geodatabase]

28. Ministry of Health, Solidarity and Promotion of Gender [Comoros] (2010). National Health Development Plan 2010-2014. Moroni, Comoros. Accessed April 4, 2017 at [http://www.nationalplanningcycles.org/sites/default/files/country_docs/Comoros/pnds_05_mai_2010_documentvf_en.pdf]

29. Ministere de la Sante (2004). Profil du Systeme de Sante. Directional National de la Sante, Ministere de la Sante, Union des Comores. Accessed September 3, 2016 at [http://tinyurl.com/z96jj6e]

30. Ministere de la sante, des affaires sociales et de la famlle [Congo] (2007). Plan National de Developpement Sanitaire 2007-2011. Brazzaville, Congo. Accessed April 4, 2017 [https://country-matrix-repository.box.com/download/external/f_434269980/0/Congo+Plan+National+de+Developpement+Sanitaire+2007-2011.pdf?shared_name=qbn28thtt7]

31. Ministry of Health and Population [2014]. Congo health facility list. Unpublished data provided by Jean-Mermoz Youndouka

32. Ministere de la sante [Côte d'Ivoire] (2012). Plan National de Developpement Sanitaire 2013-2015. Abijan, Côte d'Ivoire. Accessed April 5, 2017 http://www.nationalplanningcycles.org/sites/default/files/planning_cycle_repository/cote_divoire/pnds_2016-2020.pdf]

33. Bureau National d'Etudes Techniques et de Developpement - BNETD (2013). Projet d'Appui a la Carte Sanitaire Primaire. Rapport Final – Annexes. Unpublished excel database provided by Césaire Ahanhanzo on April 27, 2014

34. Ministere de la Sante et de la Lutte Contre le Sida (2012). Carte Sanitaire 2010 de la Cote D'ivoire. Fevrier 2012, accessed May 28, 2014 at [http://tinyurl.com/ne4hmek]

35. Ministère de la Santé Publique [Democratic Republic of Congo] (2010). Plan National de Developpement Sanitaire 2011-2015. Kinshasa, DRC. Accessed April 5, 2017 [http://www.who.int/medicines/areas/coordination/drc_pharmaceutical_profile.pdf]

36. Foundation for Innovative New Diagnostics (FIND) (2014). Mapping and characterization of health facilities: Online map of health facilities in sleeping sickness areas in the DRC. Accessed June 12, 2013 at [http://www.finddiagnostics.org/programs/hat-ond/hat/health_facilities.html]

37. Office for the Coordination of Humanitarian Affairs (2010). Structures de santé: un shapefile contenant une partie des structures de santé de la RDC. Last modified 22 September 2010 and accessed May 01, 2014 at [http://tinyurl.com/lcu3o2m]

38. Democratic Republic of Congo health facilities. Unpublished data provided by Louis Ilunga on May 01, 2014; and hospital listings per Zone de Sante provided by Ernest Dabire and Soce Fall of WHO, AFRO on June 21, 2017

39. Ministere de la sante [Djibouti] (2013). Plan National de Developpement Sanitaire 2013-2017. Djibouti, Djibouti. Accessed April 4, 2017 [http://www.nationalplanningcycles.org/sites/default/files/country_docs/Djibouti/pnds_2013_2017_partie_2_version_du_80113.pdf]

40. Ministry of Health [Djibouti] (2016). Djibouti Carte Sanitaire. Unpublished data provided by Abdisalan Mohamed Noor in Jan 2016

41. Map of hospitals in Equatorial Guinea provided by María Romay Barja and Zaida Herrador Ortiz in March 27, 2017

42. Ministry of Health [Eritrea] (2011). Health Sector Strategic Development Plan 2012-2016. Asmara, Eritrea. Accessed April 15, 2017 at [http://extwprlegs1.fao.org/docs/pdf/eri158238.pdf]

43. National Malaria Control Program (2013). Health facilities. Unpublished data provided by Selam Mihreteab on June 19, 2016

44. Ministry of Health [Ethiopia] (2015). Health Sector Transformation Plan 2012-2016. Addis Ababa, Ethiopia. Accessed April 15, 2017 at [http://www.moh.gov.et/documents/26765/0/Health+Sector+Transformation+Plan/5542a23a-9bc7-46a2-8c1f-8b32c2603208?version=1.0]

45. Ethiopia Central Statistical Agency [2011]. Rural Facilities and Services ATLAS 2011 for eight regions Accessed December 21, 2015 at [http://www.csa.gov.et/index.php/2013-02-20-14-51-24]

46. United Nations Development Program Emergencies Unit [2013]. Regional listings of health facilities used for the Expanded Programme of Immunization in Ethiopia. Unpublished data

47. Informed Decisions for Actions in Maternal and Newborn Health (2012). Data Informed Platform for Health Feasibility Study Report Amhara and Oromia Regions, Ethiopia. Accessed December 21, 2015 at [http://ideas.lshtm.ac.uk/where-we-work]

48. Ministry of Health [Ethiopia] (2013) Information for Malaria-Ethiopia facility-based survey. Unpublished data provided by the ministry of health

49. Ministere de la sante [Gabon] (2010). Plan National de Developpement Sanitaire 2011-2015. Libreville, Gabon. Accessed April 4, 2017 at

[http://csgabon.info/file/f2/Plan%20National%20de%20Developpement%20Sanitaire%20du%20Gabon%202011-2015.pdf]

50. Roll Back Malaria Central African Regional Network (2014). A list of registered health facilities in Gabon. Unpublished word database provided by Jose Nkuni on March 21, 2013

51. Ministere de la Sante, des Affaires Sociales, de la Solidarite et de la Famille (2012). Rapport d'Evaluation du Systeme d'Information Sanitaire. Fevrier 2012. Accessed 05 June, 2014 at [http://tinyurl.com/jw6vlne]

52. Ministry of Health and Social Welfare [Gambia] (2012). National Health Policy 2012-2020. Banjul, The Gambia. Accessed April 7,2017 [https://www.internationalhealthpartnership.net/fileadmin/uploads/ihp/Documents/Country_Pages/Gambia/Gambia%20National%20Health%20Policy_2012-2020%20MoHSW%5B1%5D.pdf]

53. Ministry of Health & Social Welfare [Gambia] (2015). Public Health Facilities 2014. Accessed November 11, 2015 at [http://tinyurl.com/naffp8p]

54. Synergy International (1999). Health Mapping Final Report, Gambia. Accessed November 19, 2015 at [http://tinyurl.com/pm7gkwv]

55. Ministry of Health [Ghana] (2014). Ghana Health Sector Medium Term Development Plan 2014-2017. Accra, Ghana. Accessed April 7,2017 at [http://www.moh.gov.gh/wp-content/uploads/2016/02/2014-2017-Health-sector-medium-term-dev-plan.pdf] and [http://ghanahospitals.org/home/]

56. Ghana Open Data Initiative web portal (2012). Ghana health facilities. Accessed May 6, 2016 at [http://data.gov.gh/dataset/health-facility-ghana]

57. Ministere de la sante [Guinea] (2015). Plan National de Developpement Sanitaire 2015-2024. Conakry, Guinea. Accessed April, 2017 at [http://www.nationalplanningcycles.org/sites/default/files/country_docs/Guinea/plan_national_developpement_sanitaire_2015-2024_guinee_fin.pdf]

58. Humanitarian Data Exchange (2015). Guinea: Health Centers Database. OCHA. Accessed December 7, 2015 at [http://tinyurl.com/z6588k4]

59. WHO, Guinea Bureau of Statistics & Standby Task Force (2014). Guinea Health Centers and sous prefecture 141024 DHN-SBTF. Accessed February 2, 2015 at [http://tinyurl.com/q9b46dx]

60. Ministério da Saúde Pública [Guinea Bissau]. Plano Nacional do Desenvolvimento Sanitário. Accessed April 20, 2017 at [http://www.nationalplanningcycles.org/sites/default/files/country_docs/Guinea-Bissau/pndsii_2008-2017_gb.pdf]

61. Google Earth. Hospitals in Guinea Bissau. Accessed June 27, 2017 at [https://www.google.co.ke/maps/search/hospitals+in+guinea+bissau+/@11.8235861,-15.7273989,9z/data=!3m1!4b1?hl=en]

62. Ministry of Health [Kenya] (2012). Kenya Health Sector Strategic and Investment Plan 2013-2017. Nairobi, Kenya. Accessed April 10, 2017 at [http://e-cavi.com/wp-content/uploads/2014/11/kenya-health-sector-strategic-investiment-plan-2013-to-2017.pdf]

63. Ministry of health [Kenya] (2016). Master health facility. Nairobi, Kenya. Accessed November 26, 2016 at [http://kmhfl.health.go.ke/]

64. Kenya health information system (2016). Accessed December 5, 2016 at [https://hiskenya.org/dhis-web-commons/security/login.action]

65. Noor, AM, Gikandi PW, Hay SI, Muga RO, Snow RW (2004). Creating spatially defined databases for equitable health service planning in low-income countries: the example of Kenya. *Acta Tropica*, **91:**239-251

66. Noor AM, Alegana VA, Gething PW, Snow RW (2009). A spatial national health facility database for public health sector planning in Kenya in 2008. *Int J Health Geogr.* **8**:13

67. Ministry of Health and Social Welfare [Lesotho] (2013). Health Sector Strategic Plan 2012-2017. Maseru, Lesotho. Accessed April 11, 2017 at [http://www.gov.ls/gov_webportal/important%20documents/national%20strategic%20development%20plan%20201213-201617/national%20strategic%20development%20plan%20201213-201617.pdf]

68. Christian Health Association of Lesotho (CHAL). A list of hospitals and health centers in Lesotho. Accessed at [http://www.chal.org.ls/hospitals.php]

69. Mwase, Takondwa, Eddie Kariisa, Julie Doherty, Noma phuthi Hoohlo-Khotle, Paul Kiwanuka-Mukiibi, Taylor Williamson. June 2010. Lesotho Health Systems Assessment 2010. Bethesda, MD: Health Systems 20/20, Abt Associates Inc. Accessed May 2015 at [http://healthsystemassessment.org/wp-content/uploads/2012/06/Lesotho_HSA_2010.pdf]

70. Ministry of Health and Social Welfare [Liberia] (2011). Liberia National Health and Social Welfare Policy and Plan 2011-2021. Monrovia, Liberia. Accessed April 10, 2017 at [http://www.nationalplanningcycles.org/sites/default/files/country_docs/Liberia/ndp_liberia.pdf]

71. Standby Task Force (2014). Health Facilities Liberia October 2014. Accessed June 22, 2015 at [http://tinyurl.com/zll3oyd]

72. Liberia Institute of Statistics and Geo-Information Services (LISGIS). Liberia health facilities. Accessed May 13, 2016 at [https://www.lisgis.net/index.php]

73. Ministère de la Santé Publique [Madagascar] (2015). Plan De Développement du Secteur Sante 2015-2019. Antananarivo, Madagascar. Accessed April 11, 2017 [http://www.nationalplanningcycles.org/sites/default/files/planning_cycle_repository/madagascar/pdss_2015.pdf]

74. Institute Pasteur Madagascar (2012). Madagascar health facilities. Unpublished database provided by Rakotomanana Fanjasoa and Milijaona Randrianarivelojosia on Feb 29, 2012

75. Ministry of Planning of Madagascar (2012). Madagascar health facilities. Unpublished database provided by Rakotomanana Fanjasoa and Milijaona Randrianarivelojosia on Feb 29, 2012

76. Ministry of Health [Malawi] (2011). Malawi Health Sector Strategic Plan 2011-2016. Lilongwe, Malawi. Accessed April 5, 2017 at [www.health.gov.mw/index.php/policies-strategies?download=14:malawi-health-sector-strategic-plan-2011-2016]

77. Ministry of Health & Japan International Cooperation Agency (2003). Malawi Health Facilities Inventory Survey. Lilongwe: JICA. Unpublished data provided by Adam Bennet and James Chirombo on August 21, 2012 and another on May 28, 2013

78. Standby Task Force (2014). Health Facilities Malawi October 2014. Accessed June 22, 2015 at [http://tinyurl.com/jzeloj8]

79. Malawi Health Information Systems Program (HISP). Accessed May 2016 at [http://www.hispmalawi.org.mw/dhis/dhis-web-commons/security/login.action]

80. Christian Health Association of Malawi. CHAM health facilities. Accessed June 2016 at [http://tinyurl.com/zvn4dt5]

81. Ministère de la Santé et de l'Hygiène Publique [Mali] (2014). Mali Plan Decennal de Developpement Sanitaire Et Social 2014-2023. Bamako, Mali. Accessed April 7, 2017 at [http://www.sante.gov.ml/index.php/2014-11-10-17-29-36/documents-politiques22/item/2210-plan-decennal-de-developpement-sanitaire-et-social-pddss-2014-2023]

82. MoH and UNICEF (2012). Mali health facilities. Accessed May 13, 2013 at [http://www.clustersantemali.net/docs/Carte_sanitaire_2011.pdf] with help from Massambou Sacko

83. Standby Task Force (2015). List of health facilities in Mali. Accessed December 17, 2015 at [http://tinyurl.com/j8cxtv2]

84. Ministère de la Santé [Mauritania] (2011). Plan National de Developpement Sanitaire 2012-2020. Nouakchott, Mauritania. Accessed April 7, 2017 at https://www.google.com/url?sa=t&rct=j&q=&esrc=s&source=web&cd=1&cad=rja&uact=8&ved=0ahUKEwi3obytp-XTAhWLAcAKHYmbBNAQFggpMAA&url=http%3A%2F%2Fwww.sante.gov.mr%2F%3Fwpfb_dl%3D5&usg=AFQjCNEZgCniWq3DsPOO3EgPC9inYtQ5RQ&sig2=igPHeZW1wrWFA2bvTLxDIg]

85. Ministère de la santé [Mauritania] (2014). Carte Sanitaire Nationale de la Mauritanie. Accessed April 10, 2014 at [http://tinyurl.com/j5zw8qf]

86. Ministro da Saunde [Mozambique] (2001). Mozambique Health Sector Strategic Plan 2001-2010. Maputo, Mozambique. Accessed April 7, 2017 at [www.misau.gov.mz/attachments/article/10/Plano%20Estrat%C3%A9gico%20Sectorial%20da%20Sa%C3%BAde%20-%20PESS%20-%202013-2017%20-%20Doc.pdf]

87. United Nations Children's Fund (2013). Mozambique health facilities for 10 provinces. Unpublished excel database provided by Guidion Mathe on April 15, 2013

88. Mozambique National Health Information System. Mozambique health facilities. Accessed at [http://sis-ma.in/?page_id=1327]

89. National Malaria Control Program [Mozambique] (2013). Mozambique health facilities. Unpublished data provided by Guidion Mathe

90. Ministry of Health and Social Services [Namibia] (2010). Namibia National Health Policy framework 2010-2020. Windhoek, Namibia. Accessed April 10, 2017 at [http://www.nationalplanningcycles.org/sites/default/files/country_docs/Namibia/namibia_national_health_policy_framework_2010-2020.pdf]

91. Namibia National Vector-borne Disease Control Programme (2011). Namibia health facilities. Unpublished data provided by Petrina Usuku, Bruno Moonen, John Mendhelsohn on December 5, 2012

92. Ministry of Health and Social Services (MoHSS), & ICF Macro. (2009). Namibia health facility census (HFC). Unpublished data provided by Petrina Usuku, Bruno Moonen, John Mendhelsohn on December 5, 2012

93. Clinton Health Access Initiative (2011). Namibia health facilities. Unpublished data provided by Petrina Usuku, Bruno Moonen, John Mendhelsohn on December 5, 2012

94. Ministère de la Santé Publique [Niger] (2013). Plan Strategique National de la Recherche en Sante 2013-2020. Niamey, Niger. [https://www.google.com/url?sa=t&rct=j&q=&esrc=s&source=web&cd=1&cad=rja&uact=8&ved=0ahUKEwjAtYGduNPUAhWKKcAKHQNwA8oQFggnMAA&url=https%3A%2F%2Fhealthresearchweb.org%2F%3Faction%3Ddownload%26file%3DPLAN_STRATEGIQUE_RECERCHE_EN_SANTE_2013_2020adoptjuin..pdf&usg=AFQjCNGX--2Wt59_Yf68pSqSgSgtGUuEiA]

95. World Health Organization. Niger Maps accessed on November 19, 2013 at [http://www.who.int/hac/crises/ner/maps/en/index.html]

96. Ministère de la Sante Publique (2010). Niger health facilities. Unpublished data provided by Clement Karege on November 21, 2013

97. Ministry of Health [Nigeria] (2010). National Strategic Health Development Plan 2010-2015. Abuja, Nigeria. Accessed April 10, 2017 at [http://www.health.gov.ng/doc/NSHDP.pdf]

98. Federal Ministry of Health (2012). 2011 Directory of health facilities in Nigeria. Separate Excel files provided by agreement between NMCP, sunMAP and the FMOH

99. Ministry of Health [Rwanda] (2012). Third Health Sector Strategic Plan 2012-2018. Kigali, Rwanda. Accessed April 15, 2017 at [http://www.moh.gov.rw/fileadmin/templates/Docs/HSSP_III_FINAL_VERSION.pdf]

100. National Malaria Control Programme [Rwanda] (2011). National Health Facility Database. Unpublished database provided by Dr. Corine Karema & Andrew Muhire on July 8, 2014

101. Rwanda Health Management Information System (HMIS) (2011). National Health Facility Database. Accessible online at [http://tinyurl.com/j82wr5u]

102. Ministere de la sante [São Tomé e Príncipe] (2011). Direction des soins de sante 2012-2015. Accessed April 15, 2017 at [http://www.nationalplanningcycles.org/sites/default/files/country_docs/Sao%20Tome%20and%20Principe/ppac_version_du_31-05-11.pdf]

103. Maia T, (2011). A cooperação do HFF com São Tomé e Príncipe. [PowerPoint presentation]. Accessed on July 25, 2016 at [http://tinyurl.com/hsc6wlv]

104. Ministere de la Sante et de la Prevention [Senegal] (2009). Plan National de Developpement Sanitaire 2009-2018. Dakar, Senegal. Accessed April 15, 2017 at [https://www.internationalhealthpartnership.net/fileadmin/uploads/ihp/Documents/Country_Pages/Senegal/PNDS2009_2018.pdf]

105. The United States Agency for International Development and Ministere de la Sante et de la Prevention [Senegal] (2012). Liste des structure sanitaire du Senegal 2012.

106. Ministry of Health and Sanitation [Sierra Leone] (2009). Health Sector Strategic Plan 2010-2015. Freetown, Sierra Leone. Accessed April 15, 2017 at [http://sierraleone.unfpa.org/sites/default/files/pub-pdf/nhssp_2010_15.pdf]

107. Ministry of Health and Sanitation [Sierra Leone] (2009). Sierra Leone health facilities. Unpublished data provided by Edward Magbity and Sam Smith.

108. Humanitarian Data Exchange (2015). Health facilities in Sierra Leone. Accessed December 9, 2015 at [http://tinyurl.com/oyu9q74]

109. Ministry of Human Development and Public Services [Somali] (2013) Health Sector Strategic Plan 2013-2016. Somalia. Accessed April 16, 2017 at [http://www.nationalplanningcycles.org/sites/default/files/country_docs/Somalia/the_federal_government_of_somali_republic_health_sector_strategic_plan_2013-2016.pdf]

110. Ministry of Health [Somaliland] (2013). Health Sector Strategic Plan. Somaliland, Somalia. Accessed April 16, 2017 at [http://www.nationalplanningcycles.org/sites/default/files/country_docs/Somalia/the_federal_government_of_somali_republic_health_sector_strategic_plan_2013-2016.pdf]

111. Ministry of Health [Puntland] (2013). Puntland Health Sector Strategic Plan 2013-2016. Puntland, Somalia. Accessed April 16, 2017 at [http://jhnp.org/?wpdmdl=527]

112. World Health Organisation (2013). Somalia Health Facilities. Assembled as a member of a partnership

113. United Nations Children's Fund (2013). Somalia Health Facilities. Assembled as a member of a partnership

114. OCHA (2013). Somalia - Functioning Health Facilities, April - June 2013. Accessed January 4, 2016 at [http://tinyurl.com/zsuvae2]

115. Humanitarian Data Exchange (2015). Somalia health facilities. Accessed November 3, 2016 at [https://data.humdata.org/dataset/somalia-health-facilities]

116. National Department of Health [South Africa] (2015). Health Strategic Plan 2015-2020. Accessed April 16, 2017 at [Pretoria, South Africa. http://fundisa.ac.za/wp-content/uploads/2015/10/NDOH-StrategicPlan2015-2020.pdf]

117. The National Department of Health [South Africa] (2017). South Africa health facilities. Accessed May 5, 2017 at [http://dd.dhmis.org/orgunits.html?file=NIDS%20Integrated&source=nids] with help from Ben Sartorius, Candy Day, Noluthando Ndlovu, Elizabeth Lutge and Victor Alegana

118. Ministry of Health [South Sudan] (2012). Health Sector Development Plan 2012-2016. Juba, South Sudan. Accessed April 16, 2017 at [https://extranet.who.int/nutrition/gina/sites/default/files/SSD%202012%20HEALTH%20SECTOR%20DEVELOPMENT%20PLAN%202012-2016.pdf]

119. The United Nations Office for the Coordination of Humanitarian Affairs. South Sudan: Health facilities. Accessed September 7, 2015 at [http://tinyurl.com/otm2lmp]

120. Ministry of Health [Sudan] (2003). 25 Years Strategic Plan for The Health Sector 2003-2027. Khartoum, Sudan. Accessed May 5, 2017 at [http://www.fmoh.gov.sd/English/St_Plan/doc/strategic.pdf]

121. Personal Communication Mohamed Maowia, University of Khartoum and El Fatih Malik, Federal Ministry of Health, Sudan (12th May 2016)

122. Ministry of Health [Swaziland] (2013). Health Research Strategic Plan 2014-2018. Mbabane, Swaziland. Accessed May 5, 2017 at [https://www.google.com/url?sa=t&rct=j&q=&esrc=s&source=web&cd=1&cad=rja&uact=8&ved=0ahUKEwj7kpuIteXTAhVlLZoKHQ4PCsAQFggmMAA&url=http%3A%2F%2Fwww.shrswaziland.com%2Fnotes%2FStrategic%2520Plan%2520document%2520final-Orion.doc&usg=AFQjCNGmCpI1qdIT9DqeclyScdeAvbeyoQ&sig2=By0yHBR3cEPI5mT6eg4NlA]

123. Ministry of health [Swaziland] (2010). Swaziland health facility listing of 2010. Healthcare services. Accessed June 18, 2013 at [http://tinyurl.com/jp4boc6] with help from Sabelo Dlamini and Joseph Novotny

124. Ministry of Health and Social Welfare [Tanzania] (2015). Health Sector Strategic Plan 2015-2020. Dar es Salaam, Tanzania. Accessed April 15, 2017 [http://www.tzdpg.or.tz/fileadmin/documents/dpg_internal/dpg_working_groups_clusters/cluster_2/health/Key_Sector_Documents/Induction_Pack/Final_HSSP_IV_Vs1.0_260815.pdf]

125. Ministry of Health Tanzania (2013). The Tanzania Online Health Facility Registry. Accessed March 6, 2013 at [http://hfrportal.ehealth.go.tz/] with help from Fabrizio Molteni

126. Ministre de la santé [Togo] (2012). Plan National De Developpement Sanitaire du Togo 2012-2015. Lome, Togo. Accessed April 20, 2017 at [https://www.internationalhealthpartnership.net/fileadmin/uploads/ihp/Documents/Country_Pages/Togo/PNDS_TOGO.PDF]

127. Institut National de la Statistique et des Etudes Economiques et Démographiques [Togo]. Accessed June, 2013 at [https://www.insee.fr/fr/accueil]

128. Jecker, 2013 Rapport de mission au Togo. AlterSanté. Accessed on September 21, 2016 at [http://tinyurl.com/h9osyry]

129. International Organization for Migration IOM (2014). Country Fact Sheet Togo. Accessed on September 21, 2016 at [http://tinyurl.com/zmxnx66]

130. Maman I, Badziklou K, Landoh E D, Halatoko A W, Nzussouo T N, Defang G N, Kere A B (2014). Implementation of influenza-like illness sentinel surveillance in Togo. *BMC Public Health*, ***14***: 981

131. Division de I'Information Sanitaire, Direction Generale des Etudes, de la Planification et de I'Information Sanitaire, Ministere de la Sante et de la Protection Sociale, Republique Togolaise. Lome. Accsessed on September 21, 2016 at [http://tinyurl.com/h74pwxm]

132. Ministry of Health [Uganda] (2015). Health Sector Strategic Plan III 2015-2020. Kampala, Uganda. Accessed April 15, 2017 at [https://www.google.com/url?sa=t&rct=j&q=&esrc=s&source=web&cd=1&cad=rja&uact=8&ved=0ahUKEwib_IS0t-XTAhXDQpoKHXC9AoIQFggmMAA&url=http%3A%2F%2Fhealth.go.ug%2Fdownload%2Ffile%2Ffid%2F834&usg=AFQjCNEg6ZHR2ugU5xw0XIAkLlw-P8wSjg&sig2=l7ciX_jsTj34IjHng6TFMQ]

133. Ministry of Health [Uganda] (2011). Health Facility Database. Unpublished data provided by Andrew Balyeku, Didas Namanya and Ambrose Talisuna on February 21, 2013

134. Ministry of Health [Uganda] and World Health Organization (2006). Service Availability Mapping (SAM). Accessed April 2012 at [http://www.who.int/healthinfo/systems/samdocs/en/]

135. Ministry of Health [Uganda] (2017). Master health facility list. Unpublished data provided by Lauren Hashiguchi on June 15, 2017

136. Ministry of Health [Zambia] (2011). National Health Strategic Plan 2011-2015. Lusaka, Zambia. Accessed April 15, 2017 at [http://www.moh.gov.zm/docs/nhsp.pdf]

137. Zambia Ministry of Health and World Health Organization. Service Availability Mapping Survey (2004). Unpublished data provided by John Miller and Sandra Mudhune on August 10, 2012

138. Ministry of Health and Social Welfare [Zanzibar] (2006). Health Sector Reform Strategic Plan 2007-2011. Zanzibar City, Zanzibar. Accessed April 15, 2017 at

[https://www.healthresearchweb.org/files/Zanzibar%20Health%20Sector%20Reform%20Strategic%20Plan%20II%202007-2011.pdf]

139. Health Management Information System (Zanzibar) (2013). List of Public Health Facilities in Zanzibar. Accessed July 01, 2013 at [http://41.73.201.42/hmisnews/?p=1322] with help from Rosemary Lusinde

140. Health Management Information System (HMIS) (2013). List of Public Health Facilities in Zanzibar. Accessed July 01, 2013 at [http://41.73.201.42/hmisnews/?p=132]

141. Ministry of Health and Child Care [Zimbabwe] (2016). The National Health Strategy 2016-2020. Accessed April 15, 2017 at [https://www.unicef.org/zimbabwe/National_Health_Strategy_for_Zimbabwe_2016-2020_FINAL.pdf]

142. Ministry of Health [Zimbabwe]. Zimbabwe health facilities. Accessed June 25, 2014 at [http://tinyurl.com/nahl68t]

143. The Humanitarian Data Exchange HDX (2007). Zimbabwe – Health Institutions. Accessed September 27, 2016 at [http://tinyurl.com/h9e6gve]

144. Shamu S, Training and Research Support Centre & Ministry of Health and Child Care (2014). Investigation of feasibility and options for using gap analysis in allocation of resources to support universal coverage in Zimbabwe. Harare, Zimbabwe. Accessed at [http://www.tarsc.org/publications/documents/REBUILD%20Gap%20Analysis%20Survey%20Rep%20Final%20Nov%202014.pdf]

145. World Health Organization (2009). Zimbabwe Health Cluster Bulletin No.14 16 – 31. Accessed at [http://www.who.int/hac/crises/zwe/sitreps/health_cluster_bulletin_31may2009/en/]

146. Adulai Gomes Rodrigues (2014). Sistema de Informação Hospitalar Informatizado na Guiné-Bissau. Accessed June 26, 2017 at [https://www.google.com/url?sa=t&rct=j&q=&esrc=s&source=web&cd=1&ved=0ahUKEwj9lbOK6uXUAhWEthQKHe3SCsUQFggnMAA&url=https%3A%2F%2Fsigarra.up.pt%2Fffup%2Fpt%2Fpub_geral.show_file%3Fpi_gdoc_id%3D547709&usg=AFQjCNFmbWS6fM7E4CBU6Inr_FP3zZWZHA&cad=rja]

147. Connor Catherine, Denise Averbug, and Maria Miralles. July 2010. *Angola Health System Assessment 2010.* Bethesda, MD: Health Systems 20/20, Abt Associates Inc

148. Ministry of Health [Botswana] (2010). The Essential Health Service Package. Gaborone, Botswana. April 11, 2017 at [http://www.moh.gov.bw/Publications/policies/Botswana%20EHSP%20HLSP.pdf]

149. Ministere de la sante [Burkina Faso] (2011). The health system. Accessed April 12, 2017 at [http://www.sante.gov.bf/apps/carteSanitaire/sante_bf/org_sante.htm]

150. Ministry of Health [Ethiopia] (2010). Health Sector Development Program IV 2010-2015. Addis Ababa, Ethiopia. Accessed April 15, 2017 at [https://phe-ethiopia.org/admin/uploads/attachment-721-HSDP%20IV%20Final%20Draft%2011Octoberr%202010.pdf]

151. Ghana Statistical Service (GSS), Health Research Unit, Ministry of Health, and ORC Macro. 2003. Ghana Service Provision Assessment Survey 2002. Calverton, Maryland: Ghana Statistical Service and ORC Macro. Accessed April 15, 2017 at [https://dhsprogram.com/pubs/pdf/SPA6/SPA6.pdf]

152. National Coordinating Agency for Population and Development (NCAPD) [Kenya], Ministry of Medical Services (MOMS) [Kenya], Ministry of Public Health and Sanitation (MOPHS) [Kenya], Kenya National Bureau of Statistics (KNBS) [Kenya], ICF Macro. 2011. Kenya Service Provision Assessment Survey 2010. Nairobi, Kenya: National Coordinating Agency for Population and Development, Ministry of Medical Services, Ministry of Public Health and Sanitation, Kenya National Bureau of Statistics, and ICF Macro. Accessed April 17, 2017 at [https://dhsprogram.com/pubs/pdf/SPA17/SPA17.pdf]

153. Ministry of Health and Social Services (MoHSS) [Namibia] and ICF Macro. 2010. Namibia Health Facility Census 2009. Windhoek, Namibia. MoHSS and ICF Macro. Accessed April 20, 2017 at [https://dhsprogram.com/pubs/pdf/SPA16/SPA16.pdf]

154. Ministère de la Santé Publique [Niger] (2011). Plan de developpement sanitaire 2011 – 2015. Niamey, Niger. Accessed April 15, 2017 at [http://www.who.int/pmnch/media/events/2014/nig_pds.pdf]

155. National Institute of Statistics (NIS) [Rwanda], Ministry of Health (MOH) [Rwanda], and Macro International Inc. 2008. Rwanda Service Provision Assessment Survey 2007. Calverton, Maryland, U.S.A.: NIS, MOH, and Macro International Inc. Accessed April 15, 2017 at [http://dhsprogram.com/pubs/pdf/SPA15/SPA15.pdf]

156. Ministry of Health and Social Welfare (MoHSW) [Tanzania Mainland], Ministry of Health (MoH) [Zanzibar], National Bureau of Statistics (NBS), Office of the Chief Government Statistician

(OCGS), and ICF International 2015. Tanzania Service Provision Assessment Survey (TSPA) 2014-15. Dar es Salaam, Tanzania, and Rockville, Maryland, USA: MoHSW, MoH, NBS, OCGS, and ICF International. Accessed April 23, 2017 at: [https://dhsprogram.com/pubs/pdf/SPA22/SPA22.pdf]

157. Ministry of Health [Uganda] (2014). Uganda Hospital and Health Centre IV Census Survey. Accessed April 20, 2017 at [http://health.go.ug/content/uganda-hospital-and-health-centre-iv-census-survey]

158. Ministry of Health and Social Welfare (MoHSW) [Tanzania Mainland], Ministry of Health (MoH) [Zanzibar], National Bureau of Statistics (NBS), Office of the Chief Government Statistician (OCGS), and ICF International 2015. Tanzania Service Provision Assessment Survey (TSPA) 2014-15. Dar es Salaam, Tanzania, and Rockville, Maryland, USA: MoHSW, MoH, NBS, OCGS, and ICF International. Accessed April 15, 2017 at [https://dhsprogram.com/pubs/pdf/SPA22/SPA22.pdf]
